# Discovery of the key active compounds in Citri Reticulatae Pericarpium (*Citrus reticulata* “Chachi”) and their therapeutic potential for the treatment of COVID-19 based on comparative metabolomics and network pharmacology

**DOI:** 10.3389/fphar.2022.1048926

**Published:** 2022-11-23

**Authors:** Fu Wang, Lin Chen, Hongping Chen, Zhuyun Yan, Youping Liu

**Affiliations:** Department of Pharmacy, Chengdu University of TCM, State Key Laboratory of Southwestern Chinese Medicine Resources, Chengdu, China

**Keywords:** Citri Reticulatae Pericarpium, COVID-19, Network Pharmacology, comparative metabolomics, isorhamnetin

## Abstract

Edible herbal medicines contain macro- and micronutrients and active metabolites that can take part in biochemical processes to help achieve or maintain a state of well-being. Citri Reticulatae Pericarpium (CRP) is an edible and medicinal herb used as a component of the traditional Chinese medicine (TCM) approach to treating COVID-19 in China. However, the material basis and related mechanistic research regarding this herb for the treatment of COVID-19 are still unclear. First, a wide-targeted UPLC-ESI-MS/MS-based comparative metabolomics analysis was conducted to screen for the active metabolites of CRP. Second, network pharmacology was used to uncover the initial linkages among these metabolites, their possible targets, and COVID-19. Each metabolite was then further studied *via* molecular docking with the identified potential SARS-CoV-2 targets 3CL hydrolase, host cell target angiotensin-converting enzyme II, spike protein, and RNA-dependent RNA polymerase. Finally, the most potential small molecule compound was verified by *in vitro* and *in vivo* experiments, and the mechanism of its treatment of COVID-19 was further explored. In total, 399 metabolites were identified and nine upregulated differential metabolites were screened out as potential key active metabolites, among which isorhamnetin have anti-inflammatory activity *in vitro* validation assays. In addition, the molecular docking results also showed that isorhamnetin had a good binding ability with the key targets of COVID-19. Furthermore, *in vivo* results showed that isorhamnetin could significantly reduced the lung pathological injury and inflammatory injury by regulating ATK1, EGFR, MAPK8, and MAPK14 to involve in TNF signaling pathway, PI3K-Akt signalling pathway, and T cell receptor signaling pathway. Our results indicated that isorhamnetin, as screened from CRP, may have great potential for use in the treatment of patients with COVID-19. This study has also demonstrated that comparative metabolomics combined with network pharmacology strategy could be used as an effective approach for discovering potential compounds in herbal medicines that are effective against COVID-19.

## Introduction

Coronavirus disease 2019 (COVID-19), caused by the novel coronavirus SARS-CoV-2 is a highly contagious acute respiratory disease. The main symptoms of COVID-19 are fever, fatigue, dry cough, and breathing difficulties (Alsharif and Qurashi, 2021). Additionally, a small number of patients may be ill without any accompanying symptoms. Globally, as of 8 September 2022, more than 599,000,000 confirmed cases and 6460,000 deaths have been reported to WHO. Although the development and widespread adoption of vaccines inhibited the development of the pandemic, the highly infectious delta and omicron strains has spread in many countries, putting people’s lives at risk again (Ita, 2021). Fortunately, the crystal structures of some important targets of SARS-CoV-2 have been recently resolved, providing a solid foundation for discovering and developing potential agents to control the outbreak.

Coronaviruses (CoVs) comprise a group of enveloped single-chain positive-sense RNA viruses, some of which can cause severe human respiratory diseases, including severe acute respiratory syndrome (SARS), Middle East respiratory syndrome (MERS), and the ongoing COVID-19 (Li et al., 2020). A key factor in viral infection is viral entry into host cells. For the last 20 years, the importance of endocytic pathways and the autophagy process in viral entry and replication has increasingly been recognized (Derakhshan et al., 2021; Majumder and Minko, 2021). Therefore, endocytic pathways, including endosomes and lysosomes, have become an important target for the development of treatment strategies for diseases caused by CoVs. Similar to SARS and MERS, the novel coronavirus genome encodes non-structural proteins, structural proteins, and auxiliary proteins. Among them, non-structural proteins include 3-chymotrypsin-like protease, papain-like protease, hydrocyclase, and RNA-dependent RNA polymerase. These four non-structural proteins are key enzymes in the viral life cycle. Structural proteins include the spike glycoprotein, which is essential to virus interaction with cell receptors as it enters a cell. The above five proteins are considered to be promising targets in anti-COVID-19 drug development.

Recently, network pharmacology combined with molecular docking has been used for the rapid screening of anti-COVID-19 drugs (Li R et al., 2021). In addition, machine learning has also been used to screen for anti-COVID-19 drugs (Laponogov et al., 2021). Some small molecule compounds have been reported to have potential as COVID-19 treatments. Such as remdesivir, chloroquine diphosphate, lopinavir, etc. These drugs have been widely used clinically. In addition, with the increasing research on anti-COVID-19 drugs, more and more small molecule compounds have been reported with potential activity targeting host proteins involved in viral replication or virus-derived host response (Kumar et al., 2021; Dhankhar et al., 2021). However, no specific or preventive drug has been developed so far. At present, the common clinical strategies have been drug repurposing or the use of a combination of drugs. Since the outbreak of COVID-19, traditional Chinese medicine (TCM) has played a positive role in the prevention and treatment of the disease, especially in treating cases with mild symptoms (Ren et al., 2020). One such TCM, Citri Reticulatae Pericarpium (CRP), also named as chenpi, has been included in the COVID-19 diagnosis and treatment protocol in China. It is a widely-used herbal medicine derived from plants belonging to the family *Citrus reticulata*.

CRP, an important by-product of citrus, is derived from the dried mature peel of *Citrus reticulata* Blanco and its different cultivated varieties, and is widely used by the food and drug industries (Zheng et al., 2020). The ancient records and previous studies have verified that CRP has a wide pharmacological activity (Zeng et al., 2018), including a broad-spectrum antiviral, anti-cancer, anti-oxidative, anti-aging, and anti-hypertensive activity (Idrees et al., 2021). This suggests that multiple compounds are enriched in CRP, and thus it might be a useful source for developing novel antiviral therapies for CoVs. Moreover, the ancient book of synopsis of the golden chamber recorded that CRP could treat the complications of lung diseases, such as pneumonia and asthma. Among the prescriptions for COVID-19 that have been issued in China, CRP is one of the most frequently used Chinese medicines. It has been widely used in COVID-19 prevention and treatment prescriptions and ranks second among the high-frequency Chinese medicines (Ji et al., 2022). In the prescription, it mainly plays the traditional efficacy of clearing heat and dampening phlegm. Meanwhile, recent studies also show that hesperidin, naringenin and gardenia B are the main anti-inflammatory components of CRP (Ho and Kuo, 2014). Hesperetin-7-O-glucoside in CRP also exhibits strong anti-inflammatory capacity *in vitro* and *in vivo* experiments (Wu et al., 2021). Another study showed that hesperidin may disrupt the interaction between RBD and ACE2, the human receptor for COVID-19, by binding to the receptor domain of the viral S protein (Wu et al., 2020). CRP could also exert anti-inflammatory and immune-modulating effects in severe cases of COVID-19 (Zeng et al., 2020). In conclusion, traditional ancient records, modern pharmacological studies and clinical practice have all confirmed that CRP plays an important role in the prevention and treatment of COVID-19. Therefore, exploring the potential active ingredients in CRP for COVID-19 treatment is of great significance for anti-COVID-19 drug discovery.

**FIGURE 1:**
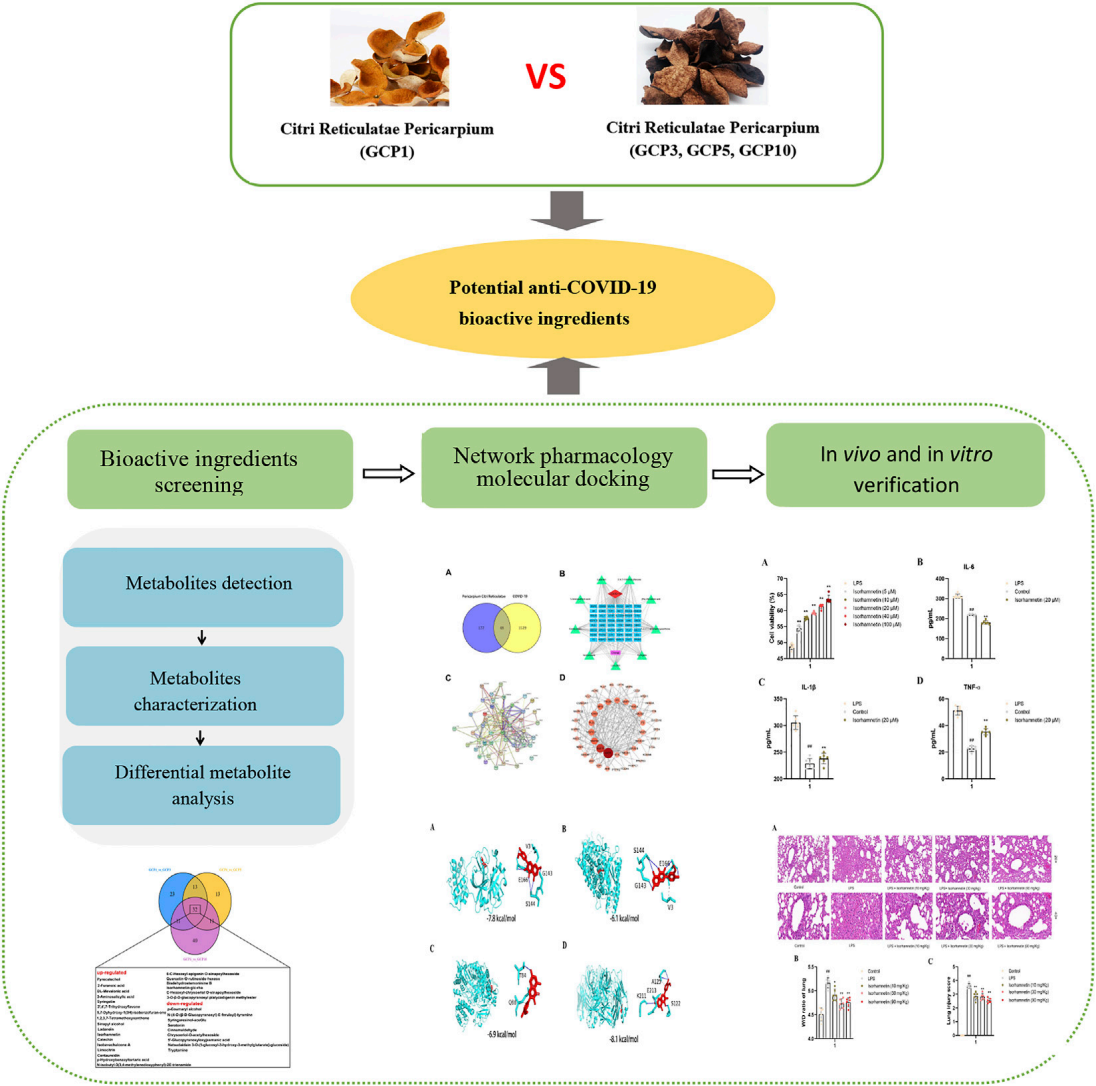
The strategy to discover potential compounds against COVID-19 based on comparative metabolomics and network pharmacology.

Historical records also mention that a longer storage period for CRP resulted in its improved medicinal effect (Yu et al., 2018; Yang F et al., 2021; Yang et al., 2022). Therefore, in this study, comparative metabolomics analysis was conducted to comprehensively characterize the chemical components present in CRP after different years of storage and discover the metabolites that may be related to its pharmacological effects. Furthermore, network pharmacology analysis was used to analysis the targets of these metabolites and COVID-19, aiming to identify the potential active metabolites of CRP may be useful as a therapy against SARS-CoV-2 replication. A molecular docking approach was also applied to link the identified metabolites to COVID-19 targets and to verify the prediction results. Lastly, the screened active compound was verified by *in vitro* and *in vivo* experiments, and the mechanism of its treatment of COVID-19 was further explored. (Figure 1). The overall objective of the study is to provide strategies for screening anti-COVID-19 components in herbal medicines.

## Materials and methods

### Materials

Twelve batches of CRP stored for different lengths of time were collected from the same place at Xinhui, Guangdong, China. Three batches were stored for 1 year (GCP1), and the other nine batches were stored for more than 1 year (GCP3, GCP5, GCP10). GCP3 = 1 < x < 3; GCP5 = 3 < x < 5; GCP10 = 5 < x < 10. X is the yeares of storage. Based on the principle that the longer the storage, the better quality of CRP, CRP stored for 1 year (GCP1) was used as the control group, and samples stored for more than 1 year (GCP3, GCP5, GCP10, respectively) were used as the experimental groups. The samples were purchased from Xin Baotang Pharmaceutical Co., Ltd. (Xinhui, China) and identified as CRP by Yan Zhuyun, an expert from the Medicinal Resources Department of Chengdu University of TCM. All information related to the batches of CRP sampled for this study is listed in Supplementary Table S1.

### Sample preparation

100 mg of each batch of the samples were weighed and dissolved in 1.0 ml methanol aqueous solution (70% methanol). The samples were sonicated for 0.5 h and placed at a low temperature for 12 h. During the period, the samples were vortex three times, centrifuged and supernatant was absorbed, filtered, and put into the injection bottle for LC-MS analysis (Li H et al., 2019).

### Ultra performance liquid chromatography and ESI-Q TRAP-MS/MS conditions

The data acquisition instrument system mainly includes Ultra Performance Liquid Chromatography (UPLC) and Tandem mass spectrometry (MS/MS). The UPLC and ESI-Q TRAP-MS/MS methods were followed by the reported methods (Table 1) (Li J et al., 2019; Xia et al., 2021).

**TABLE 1:** Liquid chromatography and mass spectrometry conditions.

UPLC conditions	Parameters	Mass conditions	Parameters
Mobile phase	A: pure water with 0.1% formic acid	Ion source temperature	500
	B: acetonitrile with 0.1% formic acid		
Elution gradient	0.00∼9.00 min, 5%∼95%B	Mass voltage	5500 v
	9.00∼10.00 min, 95%B		
	10.00–11.10 min, 95%∼5%B		
	11.10∼14.00 min, 5%B		
Flow	0.35 ml/min	Gas curtain	25 psi
Temperature	40°C	Collision induced ionization	High
Injection volume	4 μl	Ion sources and modes	Turbo spray tube
			5500 V(Positive)/−4500 V(Negative )

## Network pharmacology analys

### Screening of Citri Reticulatae Pericarpium components and target prediction

The screening criteria for all the differential metabolites were fold change values ≥ 2 or ≤ 0.5 and VIP values ≥ 1 (Yang R et al., 2021). The overlapping differential metabolites of all comparison groups were shown by Venn diagram. Among them, up-regulated metabolites were considered to be the key metabolites. The targets prediction of the key metabolites were obtained by the Traditional Chinese Medicine Systems Pharmacology (TCMSP) (Li Z. T et al., 2021; Song et al., 2021).

### COVID-19 target collection and potential target prediction

A search for genes related to the progression of COVID-19 was performed using “COVID-19” as the keyword in the GeneCards database and other database in the previous reports (Geer et al., 2010; Stelzer et al., 2016; Amberger et al., 2019; Gu et al., 2020; Ye et al., 2021).

### GO enrichment and KEGG analysis

The selected target genes were enriched by gene ontology and analyzed by Kyoto Encyclopedia of Genes and Genomes pathway using Metascape platform (Huang et al., 2009; Chen et al., 2017), and the important results are visualized by using the Weishengxin platform.

### Molecular docking

AutoDock Tools was used for molecular docking of isorhamnetin, limocitrin, syringetin, 1,2,3,7-Tetramethoxyxanthone, RS-Mevalonic acid, 7,3′,4′-Trihydroxyflavone, ladanein, 3-Aminosalicylic acid, and centaureidin with RdRp, Mpro, SP1, and ACE2 (Dhankhar et al., 2020). The hydrogen atoms and gasteiger charges were added and saved in pdbqt format. The molecular grid was set in between motif A (612–626) and motif G (499–511) covering the catalytic core of RdRp. For Mpro the grid was set in between domains 1 and 2 covering the catalytic dyad of His41 and Cys145. For SP1 and ACE2 refer to the literature method (Tao et al., 2020). The details of the grid are mentioned in Supplementary Table S6. The Lamarckian genetic algorithm was used to generate 25 docked conformations of each ligand (Singh et al., 2017). Discovery Studio (4.5 Client) software was used to perform molecular docking of the selected active components and targets, as well as dehydration of the structures. Hydrogenation and charge calculation on the proteins was then done using Autodock 4.2.6 software (Trott and Olson, 2010; Berman et al., 2019; Pinzi and Rastelli, 2019). Ebselen was docked with 3CL hydrolase, spike protein S1, angiotensin-converting enzyme II, and RNA-dependent RNA polymerase as a positive control and binding affinity was compared with isorhamnetin (Jin et al., 2020).

## *In vitro* verification of screened compound

### Cell culture and treatment

A549 cells were placed in a 37°C water bath. The cryo-storage tube was gently shaken to melt. After melting, the cells were transferred to a 15 ml centrifuge tube, and 5 ml of preheated complete medium was added slowly. The cells were collected by centrifugation, centrifuged at 1,000 rpm for 5min at room temperature, and the supernatant was discarded. The cells were suspended with complete medium, inoculated into Petri dishes, gently blown and mixed, and cultured at 37°C, 5% CO_2_ saturated humidity. When the suspended cells had grown to 80%–90%, the cells were then subcultured.

### CCK-8 assay

CCK-8 assay was used to determine the cell viability. A549 cells were inoculated into 96 well plate, and after the cells were completely adherented, different concentrations of isorhamnetin (5 μM, 10 μM, 20 μM, 40 μM and 100 μM) were added and cultured for 24 h. After 24 h of drug intervention, 10 μl of CCK-8 solution were added to each well and incubated at 37°C for 1 h. Finally, the absorbance of each well was detected at 450 nm using iMARK microplate reader (Bole, United States), and the cell viability was calculated.

### Detection of the inflammatory factors

The inflammatory factors IL-1β, IL-6 and TNF-ɑ in A549 cell supernatants were determined by kit methods.

## Animal experiments

### Experimental design

Six-week old BALB/c mices were obtained from Beijing Medical Laboratory Animal Center. The mices were injected intraperitoneally with 100 g/L chloral hydrate (4 ml/kg) and placed on a plate of 45° after anesthesia. The tongue of the mice was pulled with forceps, and 50 µl of LPS with a concentration of 1 g/L was absorbed into the posterior pharyngeal wall of the mice with a pipette gun. After 20 s, the tongue and nose were released and the mices were placed in the cage to wake up naturally. The animal experiments were approved by the Animal Ethics Committee of Chengdu University of Traditional Chinese Medicine (SYXK 2020–124).

The animals were separated into five groups (*n* = 6/group): Control group, LPS group, LPS + isorhamnetin group (10 mg/kg), LPS + isorhamnetin group (30 mg/kg), and LPS + isorhamnetin group (90 mg/kg) (Bo et al., 2022). 30 min after modeling, mices in the administration group were injected with different doses of drugs intraperitoneally. The mices were sacrificed 24 h after modeling, and the lungs were collected for detection. The control group was given corresponding normal saline.

### Histopathological evaluation

The left lobular lung tissue was cut in half, fixed with picric acid solution for 24 h, embedded in paraffin, and sectioned with 5 μm thickness. Then HE staining was performed to observe the histopathological changes of mouse lung tissue under microscope.

### Lung W/D ratio

After the mice were sacrificed, lung tissue was immediately collected and weighed. The dry weight was obtained by heating at 80°C for 48 h. Lung wet to dry ratio (W/D) was calculated by dividing lung wet weight by lung dry weight.

### Determination of inflammatory factors

An appropriate amount of lung tissue was weighed and ground with 9 times homogenization medium, then the grinding solution was centrifuged at 3000–4000 rpm for 10 min, and the supernatant was taken to prepare 10% tissue homogenate, and the levels of IL-1β, IL-6 and TNF-α were detected according to ELISA kit instructions.

### Immunofluorescence staining

Paraffin sections of 5 µ m lung tissue were prepared for IL-1 β, IL-6 and TNF-α expression by immunohistochemistry, and then observed and photographed under a ×400 magnification microscope.

### Western blot analysis

100 mg of lung tissue was put into test tube, cut into pieces, lysate was added for 1 h, centrifuged at 12,000 r/min for 15 min, and the protein concentration was measured by Bio-Rad protein quantification kit. Protein expression was detected by western blot analysis. Antibodies used herein including anti-SYK, anti-MAPK14, anti-AKT, anti-p-AKT, anti-MAPK8, anti-EGFR, anti-IL6, anti-IL-1β, anti-PIK3CA and β-actin were obtained from Affinity Biosciences LTD. The intensity of stained bands was detected with a Bio-Rad gel imaging analyzer.

### Statistical analyses

SPSS 17.0 statistical software was used for analysis. Data are expressed as mean ± standard deviation. The SNK-Q method was used for comparison between groups. A *p*-value of less than 0.05 was considered statistically significant.

## Results

### Metabolic profiling

**FIGURE 2:**
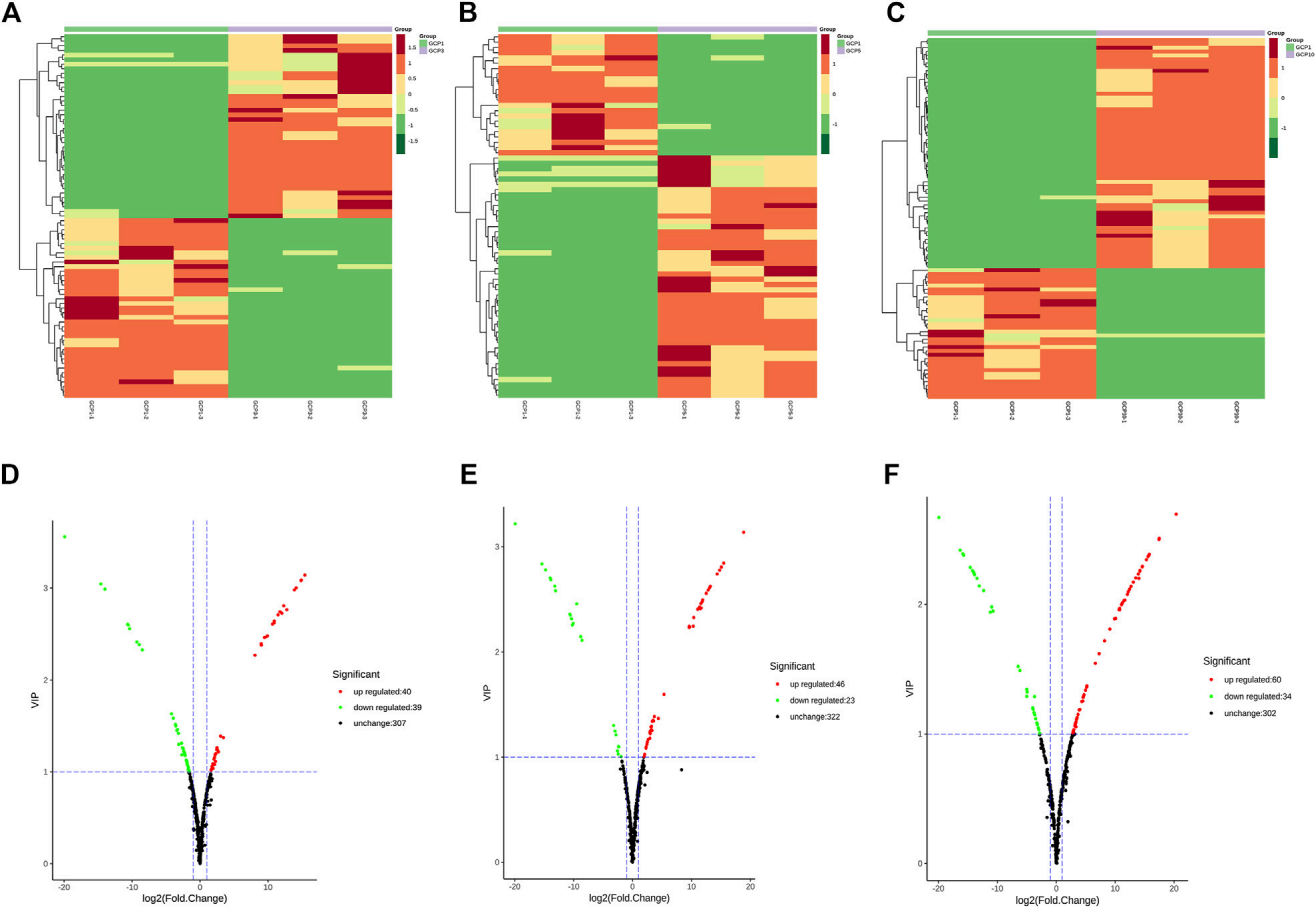
Clustering heatmap of metabolites **(A–C)** and volcano plots for the comparison group GCP1 versus GCP3 **(D)**, GCP1 versus GCP5 **(E)**, GCP1 versus GCP10 **(F)**.

In the present study, the metabolites of CRP from GCP1, GCP3, GCP5, and GCP10 were investigated based on widely targeted metabolomics. Finally, 399 metabolites were characteried in all the samples, including 6 terpenoids, 51 alkaloids, 80 phenolic acids, 4 quinones, 3 tannins, 147 flavonoids, 8 lignans and coumarins, and 90 other compounds (Supplementary Table S2). In the heatmap (Figures 2A–C), the relative content of metabolites in GCP1 was the lowest compared with GCP3, GCP5, and GCP10. Although the samples of GCP1, GCP3, GCP5, and GCP10 were grouped, the content of the metabolites was also quite different within each set. Our findings demonstrated that a longer storage time improves the metabolite content of CRP (Zheng et al., 2021).

### Analyses of differential metabolites

Principal component analysis (PCA) is a multivariate statistical method to investigate the correlation between multiple variables, and study how to reveal the internal structure of multiple variables through a few principal components. This method is often used to explore the differences between samples from different sources (Giuliani, 2017).

**FIGURE 3:**
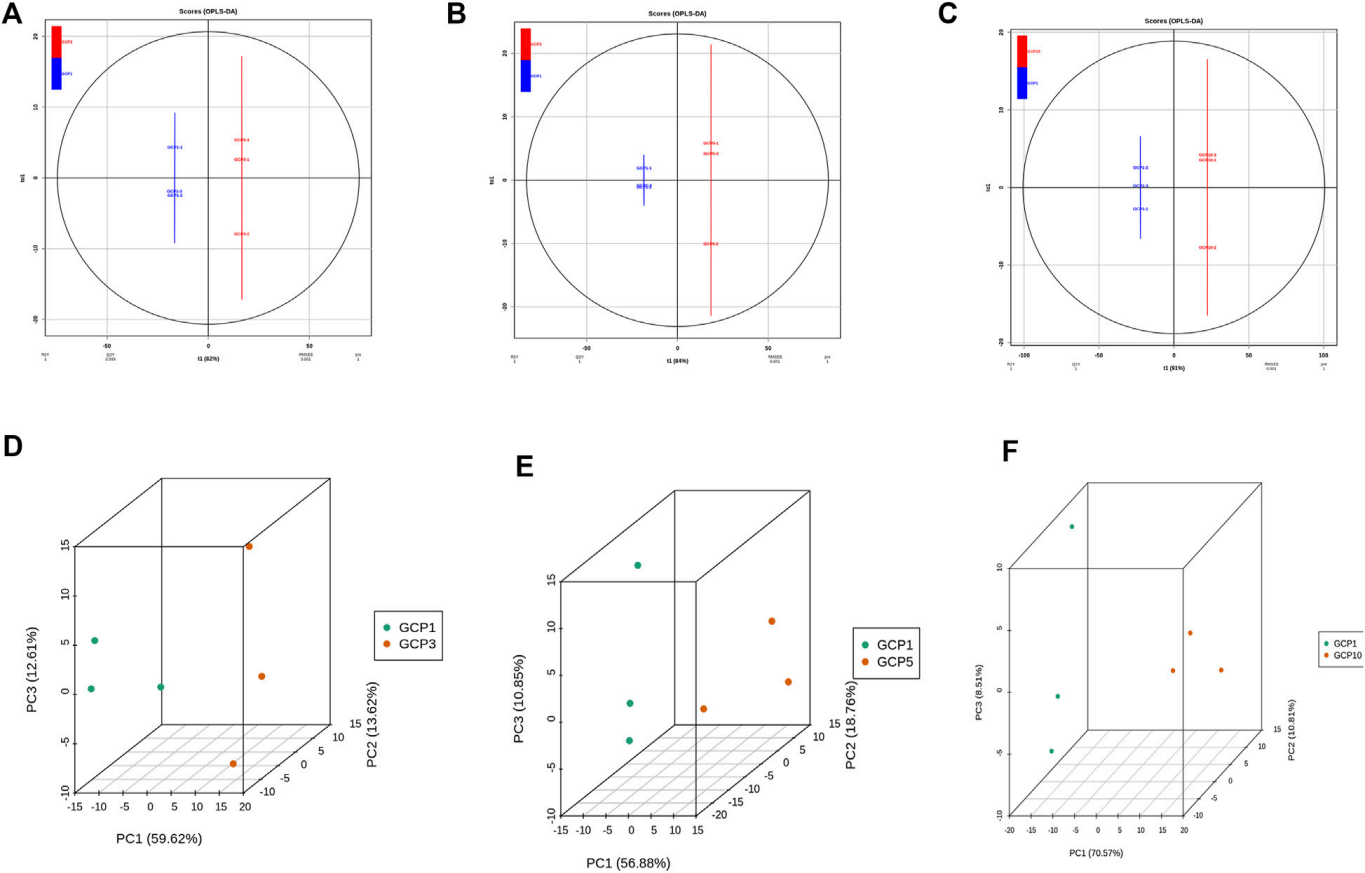
Differential metabolite analysis based on orthogonal signal correction and partial least squares-discriminant analysis (OPLS-DA) and principal component analysis for each comparison group. **(A–C)** OPLS-DA model plots for the comparison group GCP1 versus GCP3, GCP1 versus GCP5, GCP1 versus GCP10. 3D PCA plots for the comparison group GCP1 versus GCP3, GCP1 versus GCP5, GCP1 versus GCP10 **(D–F)**.

In the present study, PCA was preformed to investigate the metabolites differences of CRP with different storage years. As shown in Figures 3D–F, GCP3, GCP5, and GCP10 were clearly separated from GCP1, thus indicating that there are great differences between different samples. At the same time, the clustering of QC samples into one category also indicates the repeatability and reliability of this experiment. Similarly, orthogonal partial least squares discriminant analysis (OPLS-DA) is widely used in metabolomics analysis. Partial least squares regression is used to establish the relationship model between metabolite expression levels and sample categories, and it can also effectively separate samples and predict sample categories (Triba et al., 2015). All the differential metabolite analysis parameters of comparison groups in this paper are shown in Supplementary Tables S3–S5, including logFC, *p*-value, and VIP. Parameters of OPLS-DA model for all comparison groups in this paper are shown in Figures 3A–C. As can be seen from Figures 3A–C, the Q^2^ values of the three comparison groups were 0.999, 1,1, respectively. Since the Q^2^ value in all comparison groups was greater than 0.9, indicating that these OPLS-DA models were reliable and could be used to screen the differential metabolites in each comparison group.

A comparison between GCP1 and GCP3 showed that 40 metabolites increased and 39 decreased (Figure 2D). In the comparison between GCP1 and GCP5, 46 and 23 metabolites were up-regulated and down-regulated, respectively (Figure 2E). In the comparison between GCP1 and GCP10, 34 and 60 metabolites were down-regulated and up-regulated, respectively (Figure 2F). Notably, the number of up-regulated compounds was more than that of down-regulated compounds in all the comparison groups, which confirmed that metabolites were enriched in the longer storaged samples.

### Screening of the metabolites and potential drug targets of Citri Reticulatae Pericarpium

**FIGURE 4:**
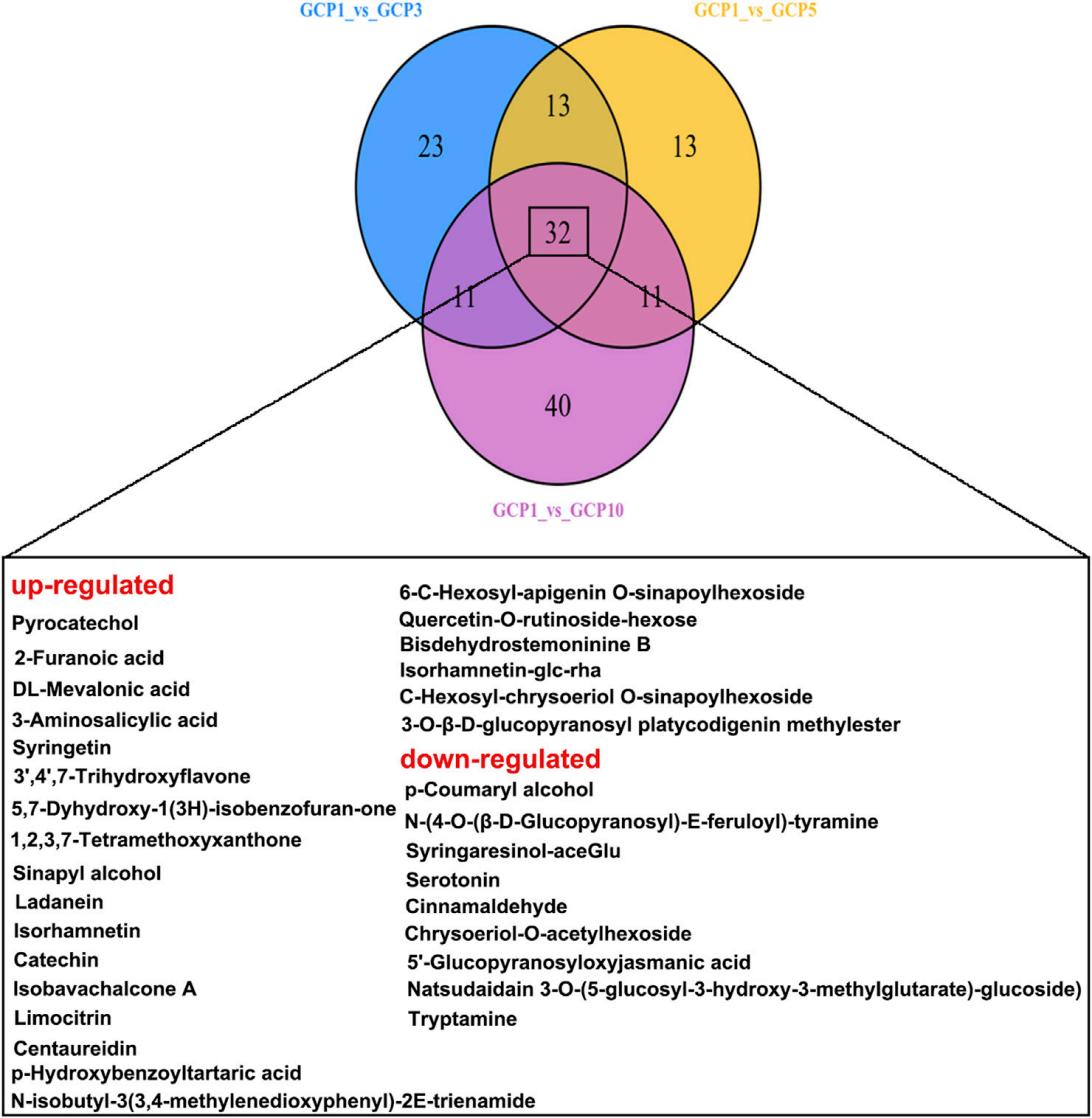
Venn diagram showing the overlapping and unique differential metabolites between the comparison groups.

**FIGURE 5:**
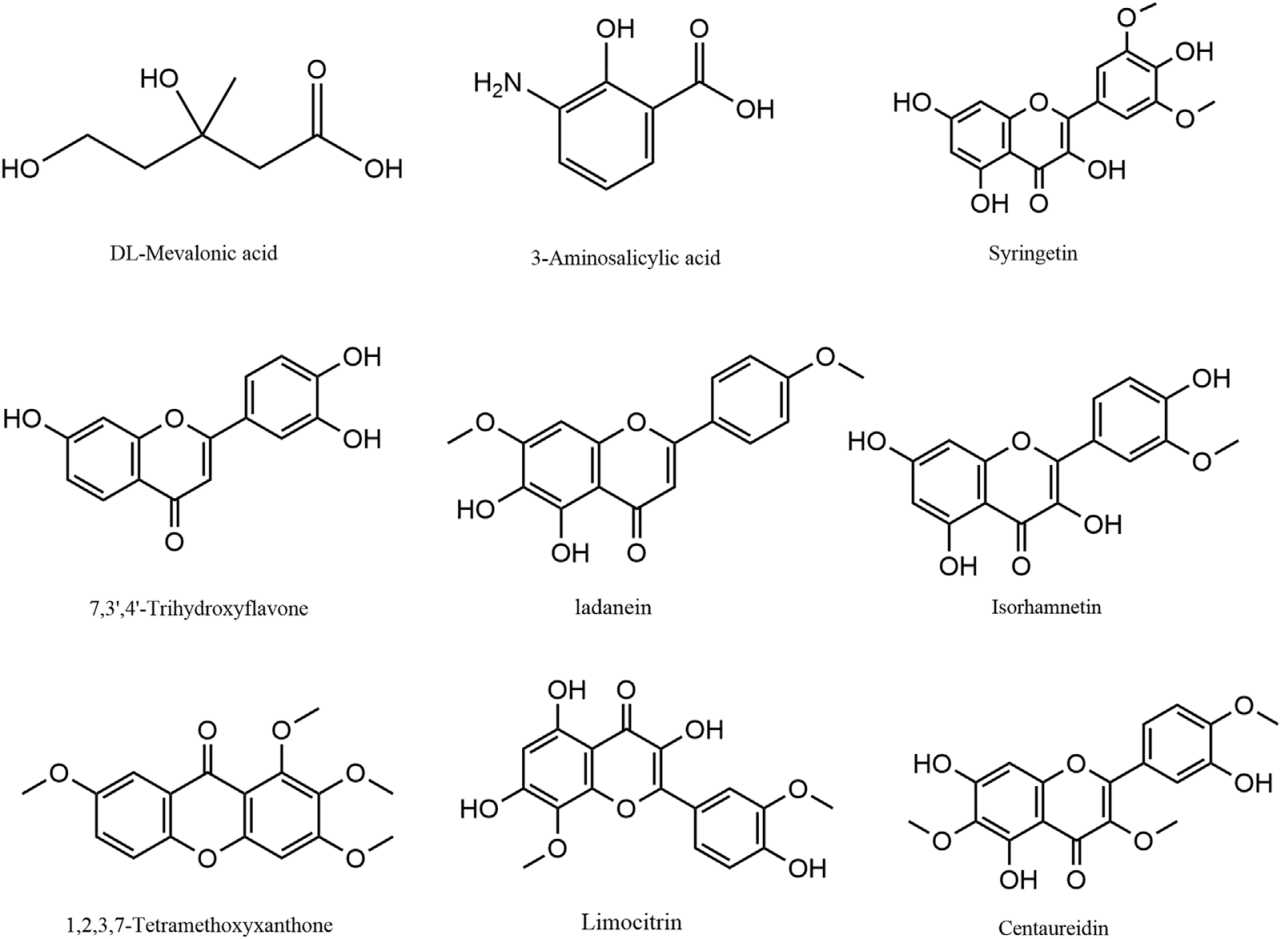
The structure of the effective ingredients in Citri Reticulatae Pericarpium.

The Venn diagram shows the differential metabolites shared by the comparison groups of GCP1 vusus GCP3, GCP1 vusus GCP5, and GCP1 vusus GCP10. From Figure 4, 32 overlapping differential metabolites were obtained. Among them, 9 of these 32 metabolites (Table 2 and Figure 5) were screened out, and they were belonging to the upregulated overlapping differential metabolites and were identified as active metabolites of CRP. Then, we identified 225 COVID-19 related targets of the above active metabolites from the TCMSP database.

**TABLE 2:** Overlapping differential up-regulated metabolites in all the comparison groups.

Components	Ions model	Formula	VIP	Fold_Change	LogFC	Type
RS-Mevalonic Acid	[M-H]-	C_6_H_12_O_4_	3.09E+00	3.06E+04	1.49E+01	up-regulated
3-Aminosalicylic acid	[M-H]-	C_7_H_7_NO_3_	1.09E+00	3.73E+00	1.90E+00	up-regulated
7,3',4'-Trihydroxyflavone	[M+H]+	C_15_H_10_O_5_	2.81E+00	5.10E+03	1.23E+01	up-regulated
Ladanein	[M+H]+	C_17_H_14_O_6_	1.37E+00	1.08E+01	3.43E+00	up-regulated
Isorhamnetin	[M-H]-	C_16_H_12_O_7_	1.22E+00	6.55E+00	2.71E+00	up-regulated
1,2,3,7-Tetramethoxyxanthone	[M+H]+	C_17_H_16_O_6_	2.48E+00	9.64E+02	9.91E+00	up-regulated
Limocitrin	M+H]+	C_17_H_14_O_8_	3.00E+00	1.82E+04	1.41E+01	up-regulated
Syringetin	[M-H]-	C_17_H_14_O_8_	1.24E+00	5.98E+00	2.58E+00	up-regulated
Centaureidin	M+H]+	C_18_H_16_O_8_	2.46E+00	7.18E+02	9.49E+00	up-regulated

### Potential targets of Citri Reticulatae Pericarpium in anti-COVID-19

We obtained 1,238, 1956 and 1567 COVID-19 targets from the NCBI, GeneCards and GenCLiP3 Databases, respectively. Finally, 1,587 potential treatment targets remained after removing duplicate targets. Using Venny 2.1 drawing software, 225 drug targets of the identified active metabolites of CRP were mapped to 1587 COVID-19-related disease targets. Finally, 48 drug targets (Figure 7A) were chosen for further analysis as potential targets in the treatment of COVID-19.

### Target network analysis of Citri Reticulatae Pericarpium

**FIGURE 6:**
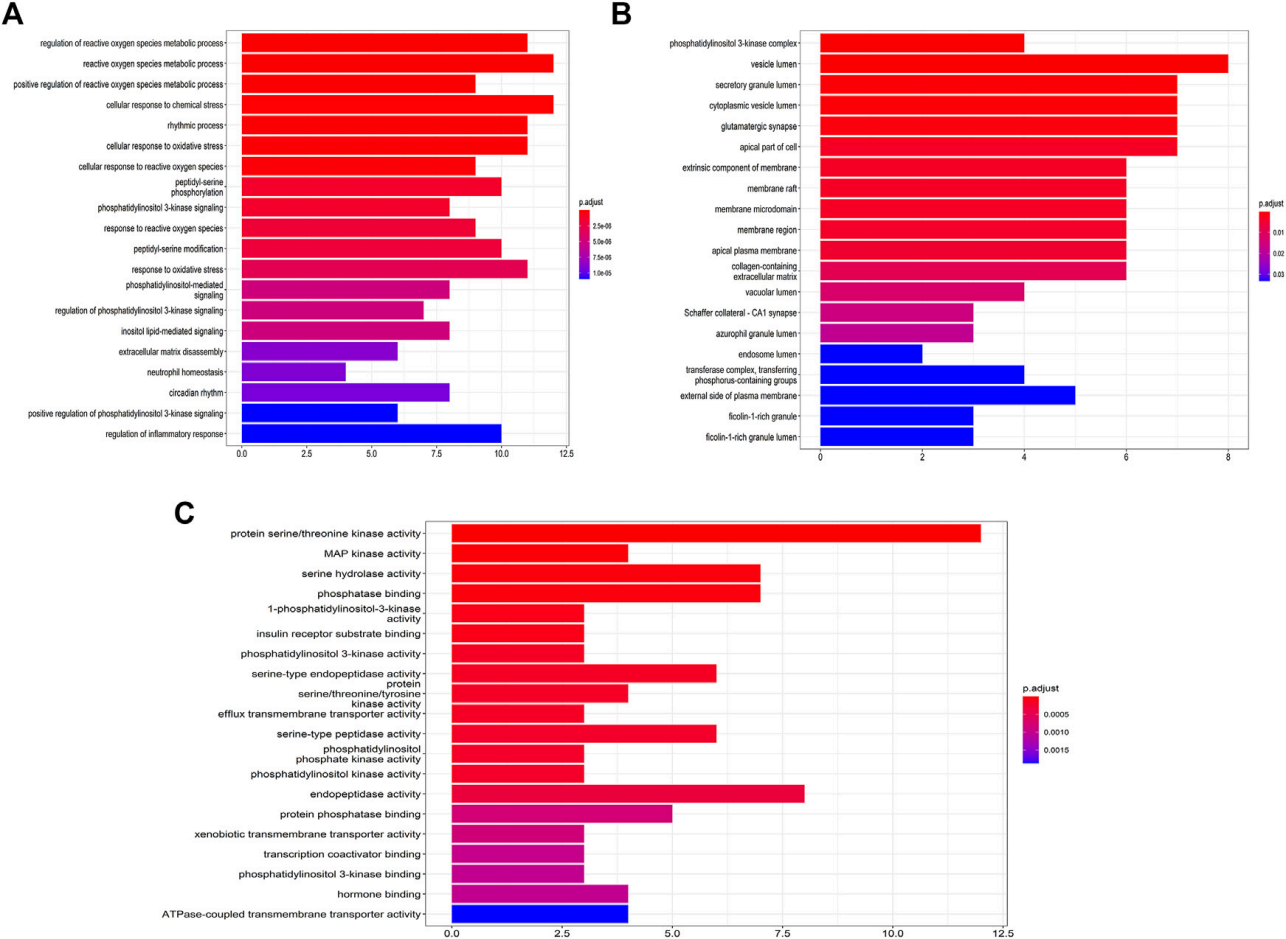
GO enrichment analysis of the intersection of Citri Reticulatae Pericarpium. **(A)** Biological process. **(B)** Cellular component. **(C)** Molecular function.

**FIGURE 7:**
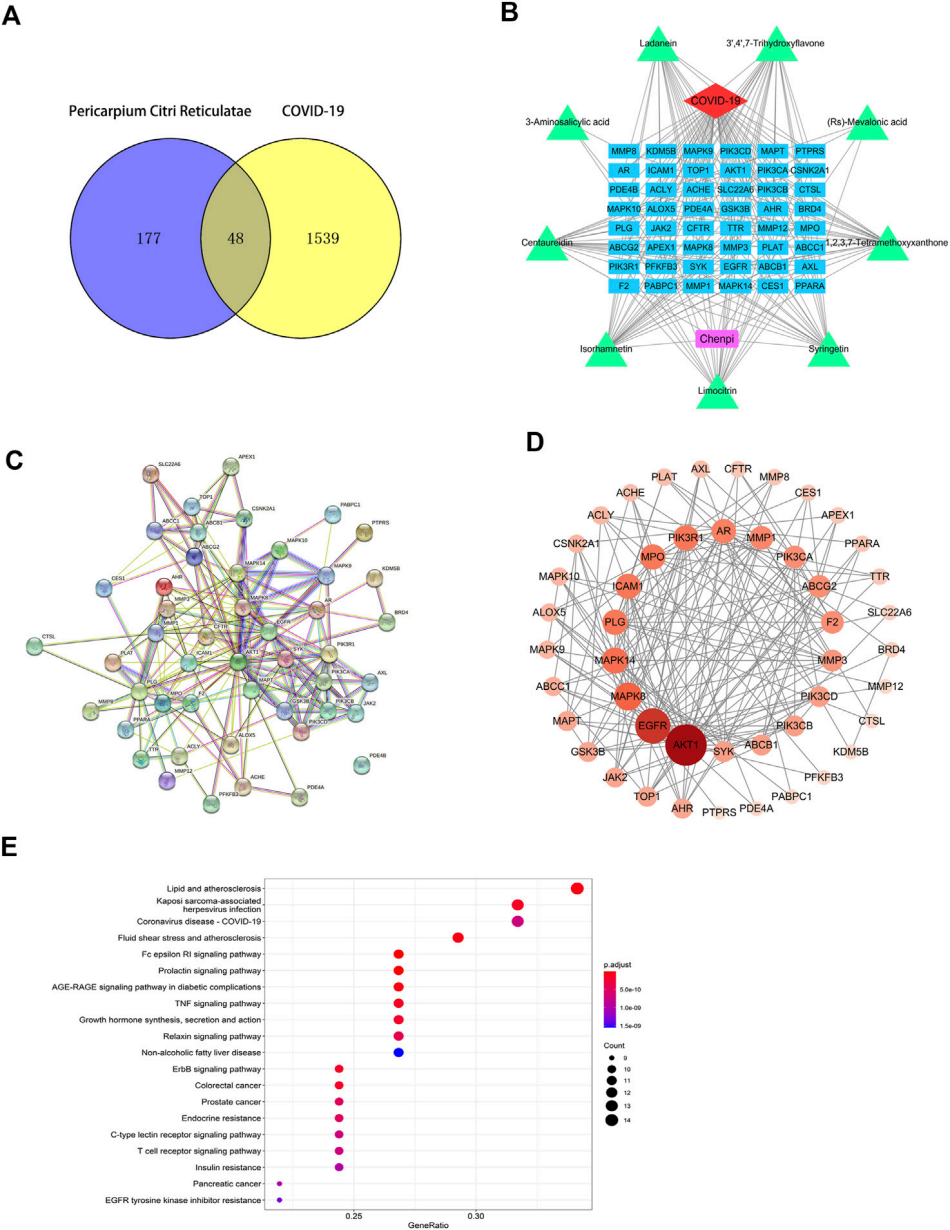
Construction of the target network of Citri Reticulatae Pericarpium. **(A)** Component-target network. **(B)** Venn diagram between constituents-related targets and COVID-19-related targets. **(C)** Network of nine constituents and overlapped COVID-19-related targets. **(D)** The PPI network. **(E)** KEGG analysis of Citri Reticulatae Pericarpium. The sizes and colors are correlated to the degrees of targets in the network: larger sizes and deeper purple colors mean a higher degree of target correlation.

Based on the data obtained, a component-target network was constructed. 48 nodes, 192 interactions make up the network (Figure 7B). Further analysis indicated that nine compounds targeted COVID-19-related targets (Figure 7C). And the genes associated with these nine compounds were constructed into a PPI network. Among them, ATK1, EGFR, MAPK8, and MAPK14 were the main targets (Figure 7D). GO enrichment analysis (Figure 6) suggested that the compounds found in CRP are mainly involved in regulating inflammatory response, oxidative stress, and the channel protein activities in host-virus interactions.

In order to further understand the relationship between the identified targets and the corresponding pathways, target-pathway interactions was constructed (Figure 7E). From the top 20 pathways (Table 3), the TNF signaling pathway, PI3K-Akt signalling pathway, and the T cell receptor signaling pathway might be the main pathways associated with these compounds in treating COVID-19.

**TABLE 3:** KEGG enrichment analysis of targets.

Description	GeneRatio	BgRatio	*p*-value	*p*-adjust	Q-value
Fcepsilon RI signaling pathway	11/41	68/8108	1.61E-14	2.08E-12	5.82E-13
Prolactin signaling pathway	11/41	70/8108	2.26E-14	2.08E-12	5.82E-13
Lipid and atherosclerosis	14/41	215/8108	1.04E-12	6.13E-11	1.72E-11
AGE-RAGE signaling pathway in diabetic complications	11/41	100/8108	1.33E-12	6.13E-11	1.72E-11
PI3K-Akt signalling pathway	12/41	139/8108	2.06E-12	7.59E-11	2.13E-11
TNF signaling pathway	11/41	112/8108	4.74E-12	1.45E-10	4.07E-11
Kaposi sarcoma-associated herpesvirus infection	13/41	194/8108	5.52E-12	1.45E-10	4.07E-11
ErbB signaling pathway	10/41	85/8108	8.02E-12	1.71E-10	4.79E-11
Colorectal cancer	10/41	86/8108	9.04E-12	1.71E-10	4.79E-11
Growth hormone synthesis, secretion and action	11/41	119/8108	9.30E-12	1.71E-10	4.79E-11
Relaxin signaling pathway	11/41	129/8108	2.27E-11	3.79E-10	1.06E-10
Prostate cancer	10/41	97/8108	3.09E-11	4.74E-10	1.33E-10
Endocrine resistance	10/41	98/8108	3.43E-11	4.85E-10	1.36E-10
Coronavirus disease - COVID-19	13/41	232/8108	5.34E-11	7.02E-10	1.97E-10
C-type lectin receptor signaling pathway	10/41	104/8108	6.26E-11	7.20E-10	2.02E-10
T cell receptor signaling pathway	10/41	104/8108	6.26E-11	7.20E-10	2.02E-10
Insulin resistance	10/41	108/8108	9.16E-11	9.67E-10	2.71E-10
Pancreatic cancer	9/41	76/8108	9.46E-11	9.67E-10	2.71E-10
EGFR tyrosine kinase inhibitor resistance	9/41	79/8108	1.35E-10	1.31E-09	3.67E-10
Non-alcoholic fatty liver disease	11/41	155/8108	1.68E-10	1.55E-09	4.34E-10

### Molecular docking of the screened components of Citri Reticulatae Pericarpium with SARS-CoV-2 3CL hydrolase, angiotensin-converting enzyme II, spike protein, and RNA-dependent RNA polymerase

In the above analyses, we showed that isorhamnetin, limocitrin, syringetin, 1,2,3,7-tetramethoxyxanthone, RS-mevalonic acid, 7,3′,4′-trihydroxyflavone, ladanein, 3-aminosalicylic acid, and centaureidin are the main active metabolites of CRP. We also identified the SARS-CoV-2 proteins 3CL hydrolase (3CL; PDB ID: 6LU7), angiotensin-converting enzyme II (ACE2; PDB ID: 1R42), spike protein (S1; PDB ID: 3BGF), and RNA-dependent RNA polymerase (RdRp; PDB ID: 6M71) as the main functional targets. Molecular docking was then performed to verify whether the active components and the targets can interact.

**FIGURE 8:**
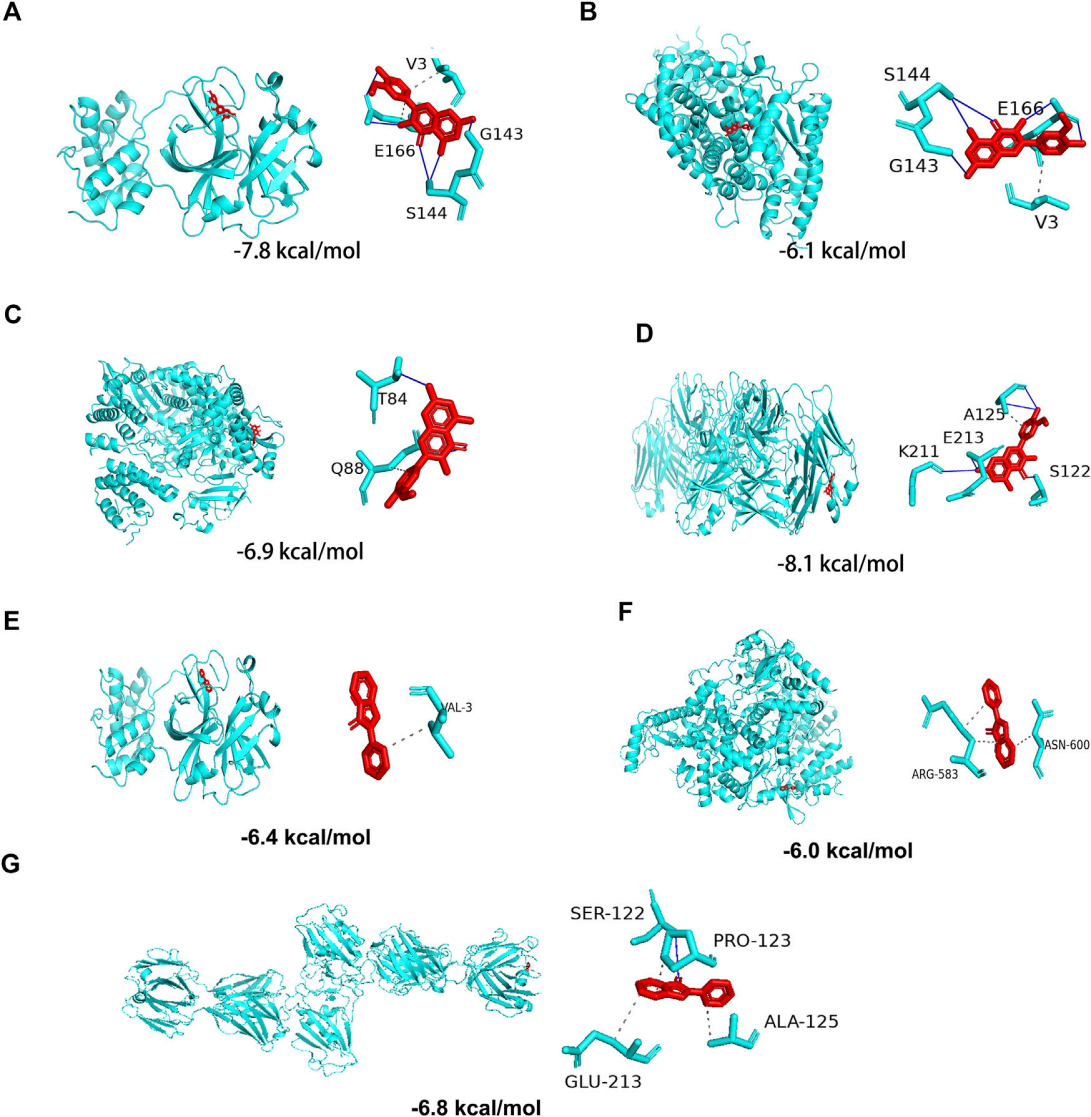
Molecular docking of the potential active Citri Reticulatae Pericarpium compound isorhamnetin with SARS-CoV-2 protein targets **(A)** 3CL, **(B)** ACE2, **(C)** S1, and **(D)** RdRp and the positive control ebselen with SARS-CoV-2 protein targets **(E)** 3CL, **(F)** RdRp, and **(G)** S1.

The molecular docking results showed that, except for RS-mevalonic acid, all the identified active metabolites of CRP had a docking score of less than −5.0 with the four main functional targets, indicating that they have strong binding activity (Table 4). Among them, the docking scores of isorhamnetin with all four targets were significantly lower than the other compounds. And the binding affinity of CRP compound isorhamnetin was also lower than the positive control ligands. This suggests a greater binding affinity and thus potentially higher biological activity. The molecular docking modes of isorhamnetin with key targets of the disease are shown in Figure 8. Molecular docking results illustrated that isorhamnetin bind with amino acid residues (G143, E166, S144, V3, T87, Q88, K211, E213, A125, S122) of RdRp, 3CL, S1, and ACE2 with a binding affinity in the range of that −8.1 to −6.1 kcal/mol. Overall, molecular docking results suggested that isorhamnetin can bind with a considerable binding affinity at the critical sites of RdRp, 3CL, S1, and ACE2.

**TABLE 4:** Molecular docking scores.

Molecule name	Docking score (kcal/mol)			
	3CL	ACE2	S1	RdRp
Isorhamnetin	−7.8	−6.1	−8.1	−6.9
Limocitrin	−6.8	−7.1	−8.1	−6.1
Syringetin	−7.0	−6.1	−6.8	−5.4
1,2,3,7-Tetramethoxyxanthone	−6.0	−5.4	−6.1	−5.0
RS-Mevalonic Acid	−5.2	−3.9	−4.4	−3.5
7,3',4'-Trihydroxyflavone	−7.5	−6.6	−6.5	−6.1
Ladanein	−7.4	−6.8	−5.9	−6.0
3-Aminosalicylic acid	−5.9	−5.0	−5.3	−5.0
Centaureidin	−6.5	−5.9	−6.0	−5.1
ebselen	−6.4	−5.7	−6.8	−6.0

### Isorhamnetin prevents LPS-induced lung inflammation *in vitro*

**FIGURE 9:**
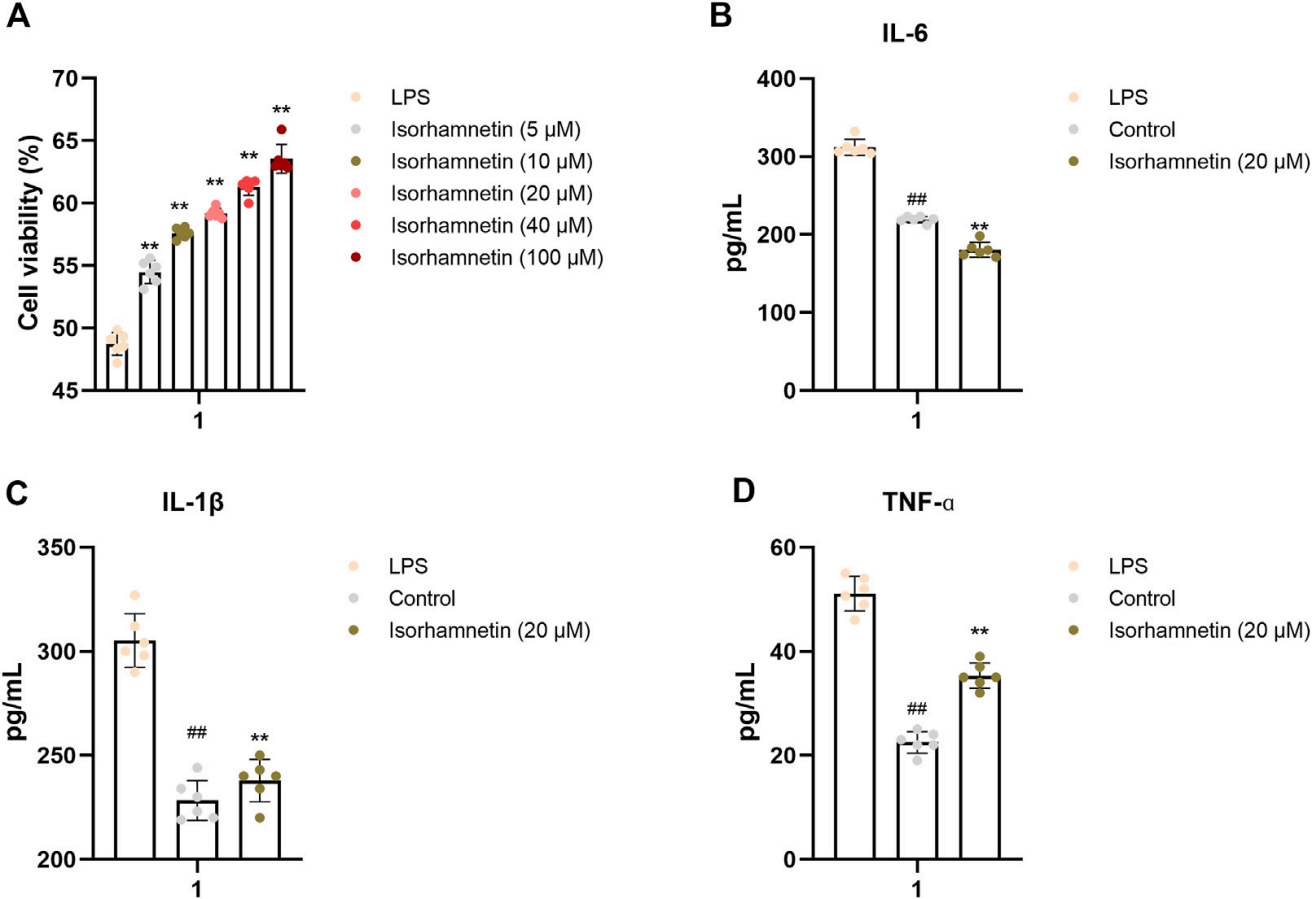
In vitro validation of isorhamnetin. **(A)** Effects of isorhamnetin on the viability of A549 cells. Effect of isorhamnetin on the production of IL-6 **(B)**, IL-1β **(C)**, TNF-ɑ **(D)** in LPS-induced A549 cells. The results represent the mean ± SEM (*n* = 3), # *p* < 0.05, ## *p* < 0.01 versus Control; *p < 0.05, **p < 0.01 cersus LPS.

CCK-8 assay was used to detect the effect of isorhamnetin on the activity of A549 cells. As shown in Figure 7, the cell activity of LPS group was significantly decreased (*p* < 0.01) compared with the blank group, indicating that LPS had a significant inhibitory effect on cell growth. Meanwhile, with the increase of isorhamnetin concentration, the cell viability was significantly enhanced, suggesting that isorhamnetin could protect A549 cells from LPS-induced injury. At the same time, the levels of inflammatory factors IL-1, IL-6, and TNF-ɑ in each treatment group were significantly lower than those in the model group (Figures 9B–D), indicating that isorhamnetin could inhibit inflammatory response in A549 cells and has anti-inflammatory activity.

### Isorhamnetin prevents LPS-induced lung inflammation *in vivo*

**FIGURE 10:**
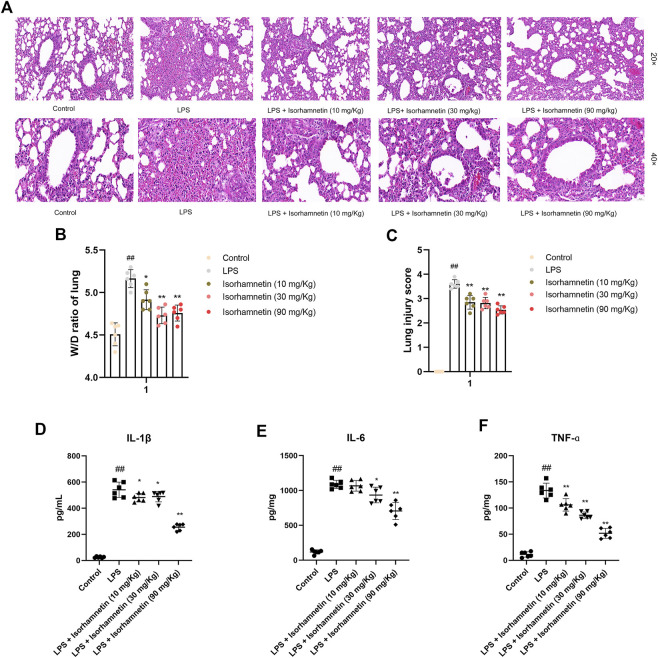
Effects of isorhamnetin on histopathological changes in lung tissues of LPS-induced lung-inflammatory mice. **(A)** HE staining of lung tissues (*n* = 6). The images were taken with eyepiece at 20× and 40×. **(B)** W/D ratio of lung tissues (*n* = 6). **(C)** Lung injury score (*n* = 6). ^#^ *p* < 0.05, ^##^ *p* < 0.01 versus Control; * *p* < 0.05, ** *p* < 0.01 versus LPS.

As shown in Figure 10A, the lung tissue in the blank group was clear, the alveolar structure was intact, there was no secretion and inflammatory cell infiltration in the alveolar cavity, and the alveolar wall thickness was normal. The lung tissue in LPS group was seriously diseased, with inflammatory cell infiltration and red blood cell exudation in alveolar cavity, thickened alveolar septum, and blood vessel congestion. Compared with LPS group, the lung tissue lesions were significantly improved, the alveolar wall was thinner, the inflammatory cells in alveolar cavity were reduced, and congestion was alleviated in the treatment group. In addition, the W/D ratio of lung and lung injury score of mices in each group were compared. The results showed that W/D ratio and lung injury were significantly decreased in the treatment group (Figures 10B,C).

### Isorhamnetin suppresses lung inflammation-related targets induced by LPS *in vivo*

**FIGURE 11:**
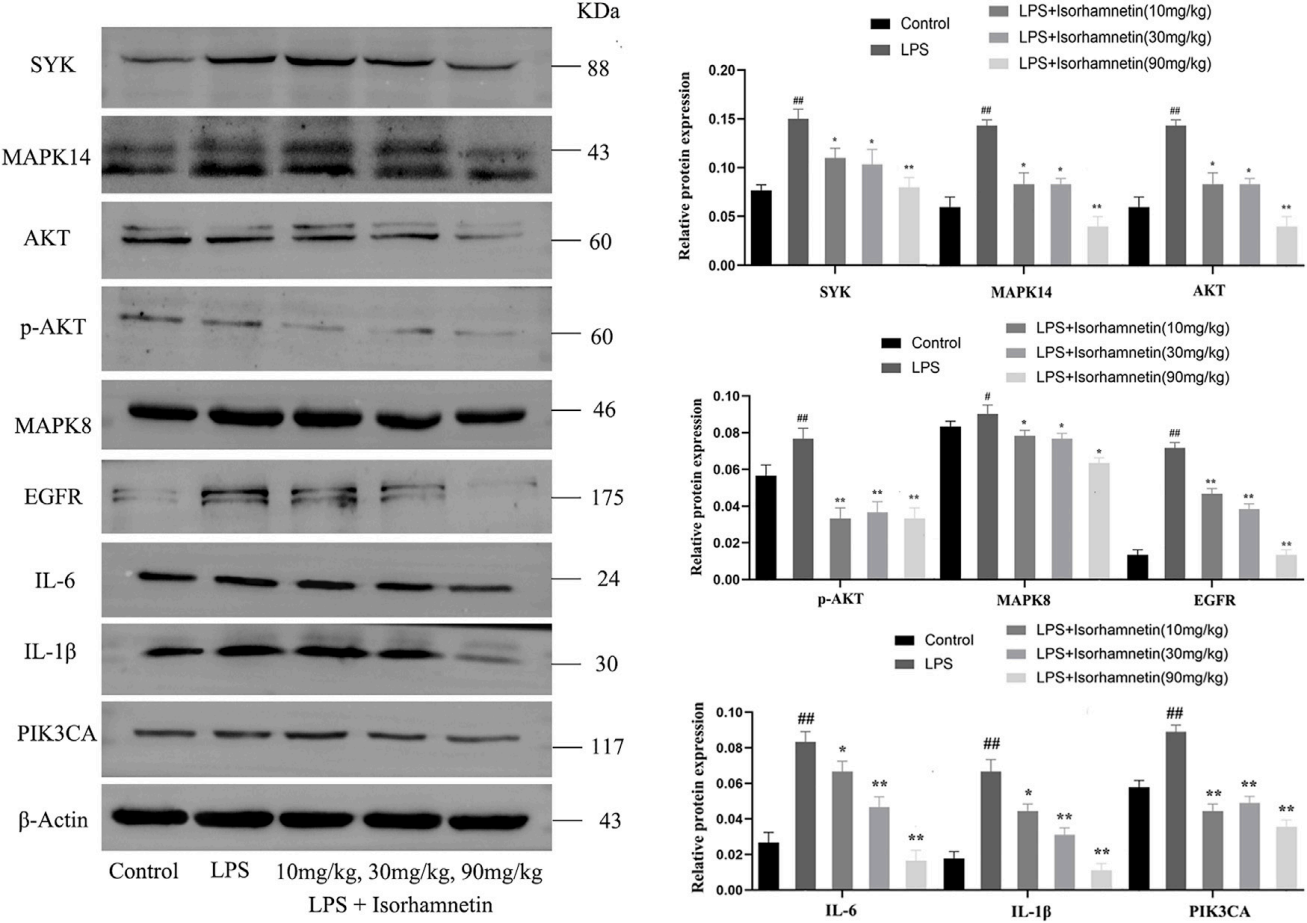
Isorhamnetin suppresses lung inflammation-related targets induced by LPS. Core target-associated markers examined by western blot (*n* = 3). # *p* < 0.05, ## *p* < 0.01 versus Control; * *p* < 0.05, ** *p* < 0.01 versus LPS.

**FIGURE 12:**
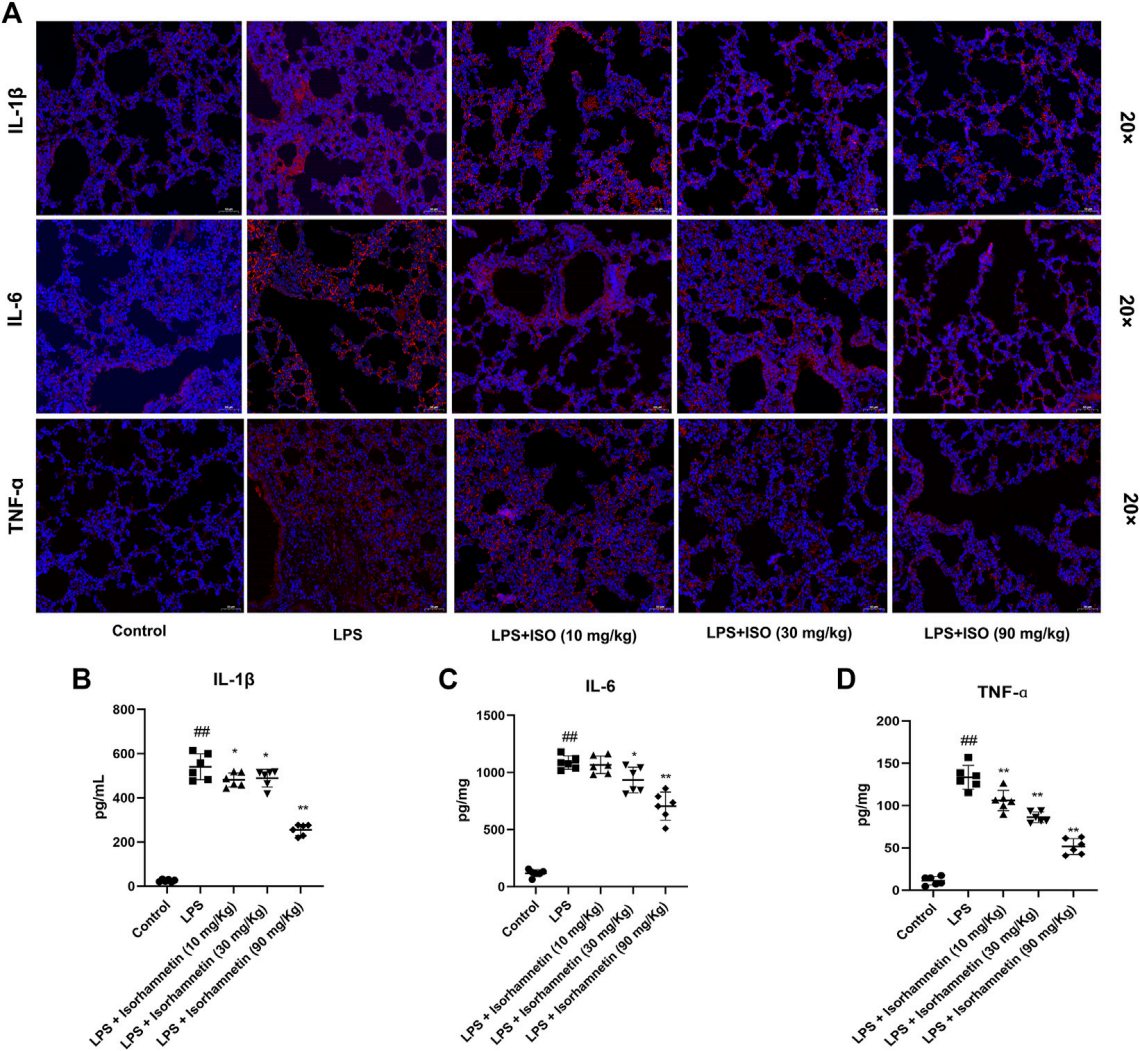
Isorhamnetin suppresses lung inflammation-related targets induced by LPS. **(A)** Core targets-associated markers examined by immunofluorescence (*n* = 3). Effect of isorhamnetin on the production of IL-6 **(B)**, IL-1β **(C)**, TNF-ɑ **(D)** in LPS-induced mice (*n* = 6). ^#^ *p* < 0.05, ^##^ *p* < 0.01 versus Control; * *p* < 0.05, ** *p* < 0.01 versus LPS.

Compared with the control group, the protein levels of SYK, IL-6, MAPK14, MAPK8, TNF-α, AKT, p-Akt, PIK3CA, EGFR and IL-1β in the model group were increased (*p* < 0.05). Compared with the model group, isorhamnetin reduced the protein expressions of SYK, IL-6, MAPK14, MAPK8, TNF-α, AKT, p-Akt, PIK3CA, EGFR and IL-1β in lung tissues of pneumonia mice (Figure 11). Immunofluorescence results also confirmed the expression of IL-1β, IL-6, and TNF-ɑ proteins (Figure 12A). In addition, isorhamnetin could also reduce the levels of inflammatory factors IL-1β, IL-6, and TNF-ɑ in lung tissue of mice in the treatment group (Figures 12B–D), suggesting that isorhamnetin inhibited the inflammatory response.

## Discussion

In the development of anti-COVID-19 drugs, the key protein targets of the virus and their crystal structures have been reported (Xu et al., 2020). Among these, the SARS-CoV-2 proteins 3CL hydrolase, ACE2, spike protein S1, and RNA-dependent RNA polymerase are the main functional protein targets of SARS-CoV-2. Based on these targets, there are currently three common strategies being studied against COVID-19. The first strategy is to test existing broad-spectrum antiviral drugs for activity against these targets (Zumla et al., 2016). Interferons, ribavirin, and cyclophilic inhibitors that were all previously used to treat coronavirus diseases fall in this category. The second strategy is to use existing molecular libraries and databases to screen molecules that may be therapeutic for the coronavirus (Gu et al., 2021). This strategy is capable of high-throughput screening of drug molecules in a very short period of time. The anti-HIV infection drug lolinovir/ritonovir also belongs to the therapies found under this category. The third strategy is the most direct: to develop new drugs from scratch based on the genomic information and pathological characteristics of CoVs. In theory, this approach would work better against CoVs. However, it would require validation of the metabolism and side effects of the developed drugs in humans and animal and human clinical trials for a long time to see if they are safe and effective (Seyed et al., 2020). Surprisingly, no comparative metabolomics approach has been performed in the search for anti-COVID-19 drugs.

Actually, the strategy of comparative metabolomics was proposed to discover potential bioactive compounds in medicinal materials by a combination of widely targeted metabolomics and network pharmacology, including sample grouping, metabolites extraction and detection, data pretreatment and quality control, metabolites identification and annotation, differential metabolites analysis, and network pharmacological prediction, which reflects not only the correlation of traditional efficacy but also the specificity and difference of the medicinal materials ingredients (Chang et al., 2020).

In this paper, we explored the potential compounds comprising CRP that may have anti-COVID-19 activity using a combined comparative metabolomics and network pharmacology approach with molecular docking and *in vitro* and *vivo* verifications. Comparative metabolomics allowed us to comprehensively characterize chemical components present in CRP samples that had been stored for different periods of time and to screen for key active metabolites. Network pharmacology has been widely used in the discovery of active compounds of drugs and traditional Chinese medicine, the explanation of the overall mechanism of action, the analysis of drug combination and prescription compatibility, etc., which provides a new idea for the study of the complex system of traditional Chinese medicine. In addition, molecular docking is a computer-based drug design technique that simulates molecular geometry and intermolecular interactions through stoichiometric calculations. That technique aims to find the best binding mode between a small molecule drug with a known structure and a large molecule (protein). Using these techniques, we hope to discover and develop potential agents to treat COVID-19.

According to clinical and pharmacological experimental design methods, CRP stored for 1 year (GCP1) was used as the control group, and samples stored for more than 1 year (GCP3, GCP5, GCP10, respectively) were used as the experimental groups. In total, 399 metabolites were identified and nine upregulated differential metabolites were screened out as potential key active metabolites of CRP. Meanwhile, six key factors in the screening of bioactive compouds in CRP were summaried. 1) The research subjects are CRP. CRP have definite medicinal history and clinical efficacy. 2) Have reasonable groupings. CRP stored for 1 year (GCP1) was used as the control group, and samples stored for more than 1 year (GCP3, GCP5, GCP10, respectively) were used as the experimental groups. 3) Have reasonable comparison. The control group and the experimental group should be arranged reasonably, and the number of up-regulated metabolites should be greater than that of down-regulated metabolites. 4) In OPLS-DA model, only Q2 exceeded 0.9, it can be used to screen differential metabolites. 5) The screening criteria of differential metabolites are fold change values ≥ 2 or ≤ 0.5 and VIP values ≥ 1.6) Those upregulated differential metabolites, both existed in all the comparision groups, act on specific targets to ameliorate a disease are regarded as potential bioactive compounds.

To further validate the effect of the screened active metabolites against COVID-19, we performed a network pharmacological analysis. The results of the KEGG pathway analysis indicated that these compounds may act on targets ATK1, EGFR, MAPK8, and MAPK14, mediating the TNF signaling pathway, PI3K-Akt signalling pathway, and T cell receptor signaling pathway. These signaling pathways are closely related to the cytokine storm caused by viral infection. Cytokine storms are prone to damage to lung epithelial cells and lung vascular endothelial cells, causing pathological changes in acute lung injury (ALI), and showing clinical representations of increased pulmonary vascular permeability and pulmonary edema (Mangalmurti and Hunter, 2020). As a result, these compounds may inhibit cytokine storm togerther through direct target action or regulation of signal pathways.

In our study, based on the results of the molecular docking, isorhamnetin, limocitrin, syringetin, 1,2,3,7-tetramethoxyxanthone, 7,3′,4′-trihydroxyflavone, ladanein, 3-aminosalicylic acid, and centaureidin both had a docking score of less than -5.0 with the main functional targets of SARS-CoV-2. Among them, isorhamnetin is among the top selective candidate to interact with drug targets, suggesting a greater binding affinity and thus potentially higher biological activity. Isorhamnetin is a known flavonoid (Zhao et al., 2019) commonly found in the flowers, leaves, and fruits of various plants such as *Ginkgo biloba* and sea buckthorn (Gong et al., 2020). It was shown to be effective in protecting vascular endothelial cells, inhibiting adipocyte differentiation, and resisting hypoxia, as well as showing anti-tumorigenic, anti-inflammatory, and antiviral properties (Zhang et al., 2020). Thus, *in vitro* and *vivo* assays were carried out to validate the therapeutic effects of isorhamnetin on lung inflammation induced by LPS. Results showed that isorhamnetin has anti-inflammatory activity *in vitro* and *vivo* assys, and could significantly reduced the lung pathological injury. In addition, other experiments, such as immunofluorescence and western blot, have also revealed that isorhamnetin could reduce the expression of inflammatory protein targets in lung tissue of mice. Therefore, isorhamnetin, as screened from CRP, may have great potential for use in the treatment of patients with COVID-19.

## Conclusion

In conclusion, we explored the potential compounds in CRP for their anti-COVID-19 activity using comparatvie metabolomics, network pharmacology, molecular docking, and verification experiments. Our results indicated that isorhamnetin screened from CRP might have potential in the treatment of COVID-19. This study has also demonstrated that the comparatvie metabolomics and network pharmacology approach could be used as an effective strategy for discovering potential compounds against COVID-19 and other diseases from herbal medicines.

## Data Availability

The original contributions presented in the study are included in the article/Supplementary Material; further inquiries can be directed to the corresponding authors.
