# Supplementary material for: Discovery of the key active compounds in Citri Reticulatae Pericarpium (Citrus reticulata “Chachi”) and their therapeutic potential for the treatment of COVID-19 based on comparative metabolomics and network pharmacology
Source: Front Pharmacol. 2022 Nov 23;13:1048926. doi: 10.3389/fphar.2022.1048926 (PMC9727096; doi:10.3389/fphar.2022.1048926)
Supplement: Supplementary file 3 [file Table1.docx]

Table S1 Smples Information

| No. | Species names | Smple sites | Sample Time | Storage time |
| --- | --- | --- | --- | --- |
| GCP1 | *Citrus reticulata* ‘Chachi’ | Guangdong Xinhui | 2020.10 | 1 year |
| GCP1 | *Citrus reticulata* ‘Chachi’ | Guangdong Xinhui | 2020.10 | 1 year |
| GCP1 | *Citrus reticulata* ‘Chachi’ | Guangdong Xinhui | 2020.10 | 1 year |
| GCP3 | *Citrus reticulata* ‘Chachi’ | Guangdong Xinhui | 2020.10 | 1~3 years |
| GCP3 | *Citrus reticulata* ‘Chachi’ | Guangdong Xinhui | 2020.10 | 1~3 years |
| GCP3 | *Citrus reticulata* ‘Chachi’ | Guangdong Xinhui | 2020.10 | 1~3 years |
| GCP5 | *Citrus reticulata* ‘Chachi’ | Guangdong Xinhui | 2020.10 | 3~5 years |
| GCP5 | *Citrus reticulata* ‘Chachi’ | Guangdong Xinhui | 2020.10 | 3~5 years |
| GCP5 | *Citrus reticulata* ‘Chachi’ | Guangdong Xinhui | 2020.10 | 3~5 years |
| GCP10 | *Citrus reticulata* ‘Chachi’ | Guangdong Xinhui | 2020.10 | 5~10 years |
| GCP10 | *Citrus reticulata* ‘Chachi’ | Guangdong Xinhui | 2020.10 | 5~10 years |
| GCP10 | *Citrus reticulata* ‘Chachi’ | Guangdong Xinhui | 2020.10 | 5~10 years |
