# Supplementary material for: Discovery of the key active compounds in Citri Reticulatae Pericarpium (Citrus reticulata “Chachi”) and their therapeutic potential for the treatment of COVID-19 based on comparative metabolomics and network pharmacology
Source: Front Pharmacol. 2022 Nov 23;13:1048926. doi: 10.3389/fphar.2022.1048926 (PMC9727096; doi:10.3389/fphar.2022.1048926)
Supplement: Supplementary file 5 [file Table6.docx]

Table S6. The details of molecular grid of SARS-CoV2 RNA-dependent RNA polymerase (RdRp), 3CL hydrolase (3CL), spike protein (S1), and angiotensin-converting enzyme II (ACE2) for molecular

| No. | Protein | AutoDock Tools Grid details | | |
| --- | --- | --- | --- | --- |
|  |  | Center grid  Points | Grid Size | Grid Point Spacing |
| 1 | RdRp | x=-14.75,  y=17.87,  z=67.18 | x = y = z = 100 | 0.30 Å |
| 2 | 3CL | x=119.33,  y=114.66,  z=134.11 | x = y = z = 100 | 0.30 Å |
| 3 | S1 | x=-0.34,  y=35.01,  z=100.22 | x = y = z = 30 | 0.35 Å |
| 4 | ACE2 | x=-39.24,  y=84.01,  z=46.44 | x = y = z = 11 | 0.35 Å |
